# Non-invasive visualization of the complete cardiac conduction system using magnetic resonance microscopy

**DOI:** 10.1186/1532-429X-13-S1-M1

**Published:** 2011-02-02

**Authors:** Min-Sig Hwang, Katja E Odening, Bum-Rak Choi, Gideon Koren, Stephen J Blackband, John R Forder

**Affiliations:** 1 McKnight Brain Institute, University of Florida, Gainesville, FL, USA; 2 Cardiovascular Research Center, The Rhode Island Hospital, Alpert Medical School of Brown University, Providence, RI, USA

## Background

The impulse-conducting system that coordinates the cardiac cycle requires a well-orchestrated interplay of its multiple heterogeneous components for synchronous and reliable contractions [1]. Thus, detailed insight into the anatomy of the conduction system would be of great significance to understand the cardiac performance arising from electro-mechanical activity [2,3]. To date, the techniques most commonly used to study the conduction system are invasive, and use intrinsically destructive procedures. MR imaging has previously used to visualize free-running Purkinje fibers[3,4], but not those in the myocardium, and thus only provide a partial account of the conduction system.

## Purpose

In this study, we aim to provide non-invasive MR data suitable to describe the complete conduction system and anatomical features in isolated rabbit hearts, as a precursor to developing a mathematical model of depolarization in the heart.

## Materials and method

Hearts (n = 3) of New Zealand White male rabbits (2-4 kg) were isolated and fixed *in situ* according to the approved animal protocol.

### MRI

MR experiments were performed on a 17.6 T / 89 mm vertical wide-bore magnet (Bruker Instruments, Billerica, MA). Three dimensional MR microscopy data were collected using a fast gradient pulse sequence, achieving a voxel resolution of 35 x 35 x 82 μm^3^. High angular resolution diffusion microscopy (HARDM) was performed with a standard PGSE sequence, achieving an in-plane resolution of 60 μm^2^ with a slice thickness of 600 μm. The b-value was 1000 s/mm^2^.

### Data analysis

Volume rendering of the 3D MR data sets was performed using ImageJ (ver. 1.31, http://rsbweb.nih.gov/ij/). The tensor processing of HARDM data sets was conducted using fanDTasia™ (©2008, http://www.cise.ufl.edu/~abarmpou/ ).

## Results

**Figure 1:**
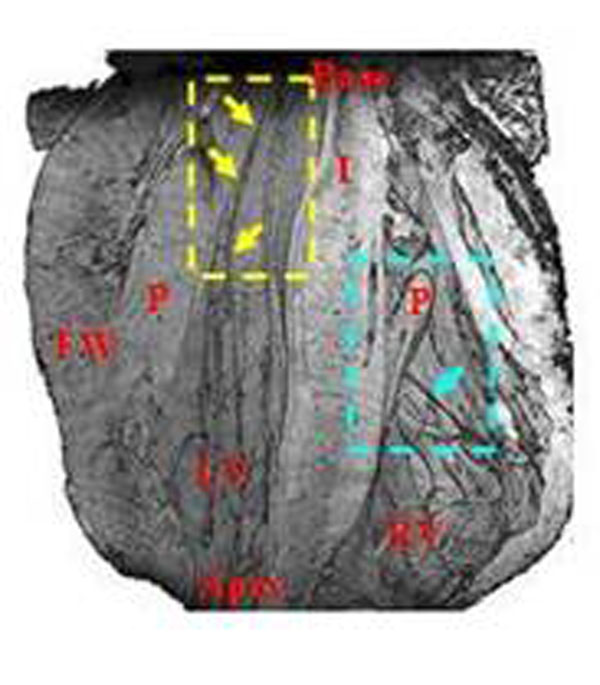
A representative volume rendered 3D MR image to visualize the conduction paths and anatomical features in an isolated heart. A red line and a green arrow in a red box indicate where sectioning occurred and viewer was located to perform the volume rendering. Note that free-running Purkinje fibers in the left ventricular cavities, a left bundle branch (yellow arrows) in ventricular inner wall, a right bundle branch (cyan arrow) and right bundle (right, yellow circle). LV: left ventricular cavity, RV: right ventricular cavity, leaflets of the mitral valve, FW: free wall, P: papillary muscle, I: ventricular interseptum.

**Figure 2:**
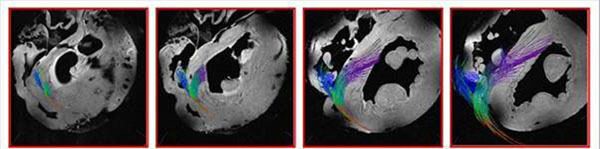
Representative data set from rabbit heart showing fiber tracking of the conduction network from base to midwall (A → D). The conducting network originates behind the non-coronary leaflet at the base (A) and quickly bifurcates (B) into fibers that proceed down the right ventricular subendocardium (shown in blue) and a branch that continues along the septum for a short distance before continuing down the left ventricular subendocardium (C, D) in a fan-like structure (shown in purple). The fan-like structure terminates in free-running Purkinje fibers in the left ventricle. Data from conducting fibers that are more apical has been omitted for clarity.

**Figure 3:**
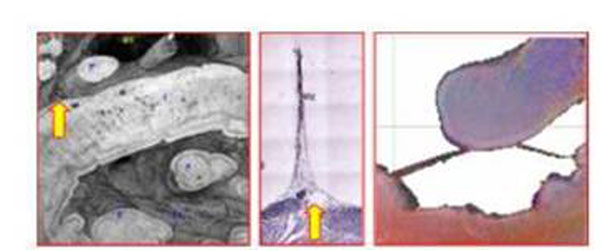
Representative images of the free-running Purkinje fibers that show septal attachment from the ventricular cavities. A volume rendered image (left), neurofilament stained image (middle), and the primary eigen vector map (right).

**Figure 4:**
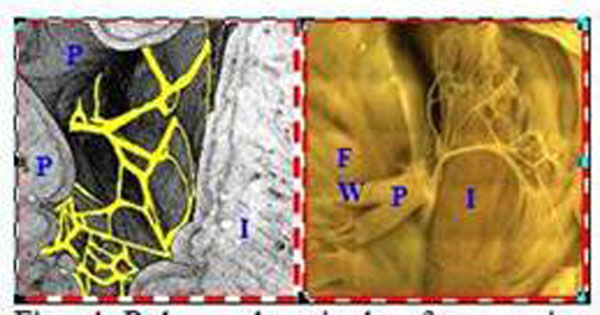
Polygonal reticular free-running Purkinje fiber network in the LV cavity. A magnified volume rendered MR image (left, yellow is manually segmented with visual inspection) and an optical image of Acetylecholine esterase staining (right, unfolded).

Figure 1 shows that a volume rendered image from the original 3D MR data made it possible to non-invasively and reproducibly trace the conduction paths in both ventricles, as well as to describe the micro-anatomical features of the heart. Figure 2 demonstrates that fiber tracking from the HARDM data sets represents the conducting pathways from the connecting bundle to the left/right bundles. Since the tendinous fiber-like cords observed in the ventricular cavities contain conducting fibers, called the free-running Purkinje fibers, the primary eigenvector of the cords may correspond to the neurofilament stained Purkinje fibers (Fig. 3). The free-running Purkinje fibers form a reticular polygonal net in the ventricular cavities (Fig. 4). In conclusion, these results demonstrate that MR microscopy is an especially promising modality for complete visualization of the conduction pathways.
